# Protein phosphatase PHLPP induces cell apoptosis and exerts anticancer activity by inhibiting Survivin phosphorylation and nuclear export in gallbladder cancer

**DOI:** 10.18632/oncotarget.3721

**Published:** 2015-03-30

**Authors:** Yinghe Qiu, Xiaoya Li, Bin Yi, Junnian Zheng, Zhangxiao Peng, Zhihan Zhang, Mengchao Wu, Feng Shen, Changqing Su

**Affiliations:** ^1^ Department of Molecular Oncology & Biliary Tract Surgery, Eastern Hepatobiliary Surgery Hospital & National Center of Liver Cancer, Second Military Medical University, Shanghai, China; ^2^ Jiangsu Center for the Collaboration and Innovation of Cancer Biotherapy, Cancer Institute, Xuzhou Medical College, Xuzhou, Jiangsu, China

**Keywords:** gallbladder carcinoma, protein phosphatase, survivin, apoptosis, cell signaling

## Abstract

Many factors regulate cancer cell apoptosis, among which Survivin has a strong anti-apoptotic effect and PHLPP is a tumor suppressor gene that can induce significant apoptosis. However, the relationship between PHLPP and Survivin in gallbladder carcinoma (GBC) has not been reported. This study found that PHLPP expression is decreased and Survivin expression is increased in GBC tissues and cell lines. Their expression levels showed an inverse relationship and were associated with poor prognosis of GBC patients. Loss of PHLPP can increase the level of phosphorylated Survivin and induce the nuclear export of Survivin, which thus inhibit cell apoptosis and promote cell proliferation in GBC cells. The process that PHLPP regulates Survivin phosphorylation and intracellular localization is involved in AKT activity. Re-overexpression of PHLPP in GBC cells can decrease AKT phosphorylation level. Reduced expression of PHLPP in GBC is associated with high expression of miR-495. Increasing PHLPP expression or inhibiting miR-495 expression can induce apoptosis and suppress tumor growth in GBC xenograft model in nude mice. The results revealed the role and mechanism of PHLPP and Survivin in GBC cells and proposed strategies for gene therapies targeting the miR-495 / PHLPP / AKT / Survivin regulatory pathway.

## INTRODUCTION

Inhibition of apoptosis is a main factor in the uncontrolled proliferation of cancer cells and is closely related to the tumorigenesis and progression of malignant tumors. There are many factors affecting the apoptosis and proliferation of cancer cells. In most types of human tumors, such as breast, liver, lung, colorectal, pancreatic, stomach cancers and malignant melanoma, activation of extracellular signal regulated kinase (ERK) and phosphatidylinositol-3-kinase (PI3K)/protein kinase B (AKT) signaling pathways induces changes in biological behaviors, such as malignant transformation, inhibition of apoptosis, increased activity of cell proliferation and metastasis. These effects result in malignant progression of cancer and poor prognosis of patients [1-3]. Src and Ras oncogenes inhibit apoptosis of gallbladder carcinoma (GBC) cells by activating the epidermal growth factor receptor (EGFR) signaling pathway [4]. Activation of the STAT3 signaling pathway can upregulate the target genes, including Bcl-X, Bcl-2, Cyclin D1, vascular endothelial growth factors (VEGF), Fas, Survivin and other factors, allowing cancer cells to escape apoptosis and inducing cell proliferation [5, 6]. Among these anti-apoptotic factors, Survivin is the most potent inhibitor of apoptosis ever found. Survivin belongs to the family of inhibitor of apoptosis proteins (IAP) and is involved in various aspects of embryonic development and cell cycle regulation. It plays dual roles in inhibiting apoptosis and regulating cell proliferation. Survivin overexpression inhibits apoptosis through various mechanisms and promotes abnormal cell proliferation and malignant transformation [7-10]. Survivin expresses in embryonic tissues and most tumor tissues but not in normal mature tissues. Survivin expression in malignant tumors is highly selective and is associated with high proliferative activity, recurrence, metastasis, resistance to radiotherapy and chemotherapy, and poor prognosis of patients. As such, Survivin is a prognostic marker of a broad spectrum of tumors and an effective molecular target for therapeutics [10, 11]. However, the regulatory mechanism of Survivin overexpression in malignant tumors is not clear yet.

In contrast, there are factors that can induce apoptosis and inhibit cancer cell proliferation. The phosphatase and tensin homolog deleted on chromosome ten (PTEN) and the tumor suppressor gene p53, both of which are tumor suppressor genes essential to the regulations of development and cell proliferation, are considered “Gene Keepers” [12]. PTEN can regulate the transcriptional activity of p53 gene and the expression level of p53 protein through the phosphatase-dependent and phosphatase-independent pathways, therefore promoting apoptosis and inhibiting cell proliferation [13]. The cytokine IL-24 can induce cancer cell apoptosis by activating caspase-9, caspase-3 and poly ADP-ribose polymerase (PARP) through downregulation of Bcl-2 expression and induction of cytochrome C release [14]. Recently, PH domain leucine-rich repeat protein phosphatase (PHLPP) has been found to have a strong apoptosis-inducing function since being discovered in 2005. On the one hand, PHLPP can mediate dephosphorylation of AKT, protein kinase C (PKC) and S6 kinase, leading to the inactivation of these factors and the inhibition of signal transduction pathways related to these protein kinases. On the other hand, PHLPP can promote cell apoptosis by inducing dephosphorylation of pro-apoptotic kinase Mst1. These two functions act together to mediate significant pro-apoptotic and growth inhibition effects in tumor cells [15-17]. PHLPP is considered as a tumor suppressor gene and can inhibit tumorigenesis, cancer progression, invasion and metastasis [18]. However, PHLPP expression is often decreased or absent in a variety of tumors [19]. The cause of decreased PHLPP expression in tumor tissues is unknown. In addition, the regulatory mechanism of PHLPP in GBC proliferation and metastasis has not been reported.

As aforementioned, Survivin is a factor with strong anti-apoptotic effect. PHLPP is a tumor suppressor gene that can induce significant apoptosis. It is worthwhile to further study the physiological and pathological functions and molecular regulatory mechanism of these two factors in tumorigenesis and tumor progression. Through experimental observation of GBC clinical specimens and cell lines, we found that the expression of PHLPP is decreased in tumor tissues and cell lines of GBC, whereas the expression of Survivin is increased. Both of these changes are associated with high malignancy of GBC and poor prognosis of patients. PHLPP is an upstream regulator of Survivin. PHLPP downregulates AKT activity and inhibits Survivin phosphorylation and nuclear export, thereby inducing apoptosis in GBC cells. The reduced expression of PHLPP in GBC cells is associated with high expression of miR-495. This study reveals the role and mechanism of PHLPP and Survivin in GBC cells and proposes strategies for gene therapies targeting the miR-495/PHLPP/AKT/Survivin regulatory pathway, which have important application prospects in the comprehensive treatment of GBC.

## RESULTS

### Decreased PHLPP and increased Survivin are closely related to the progression-free survival of GBC patients after surgery

**Figure 1:**
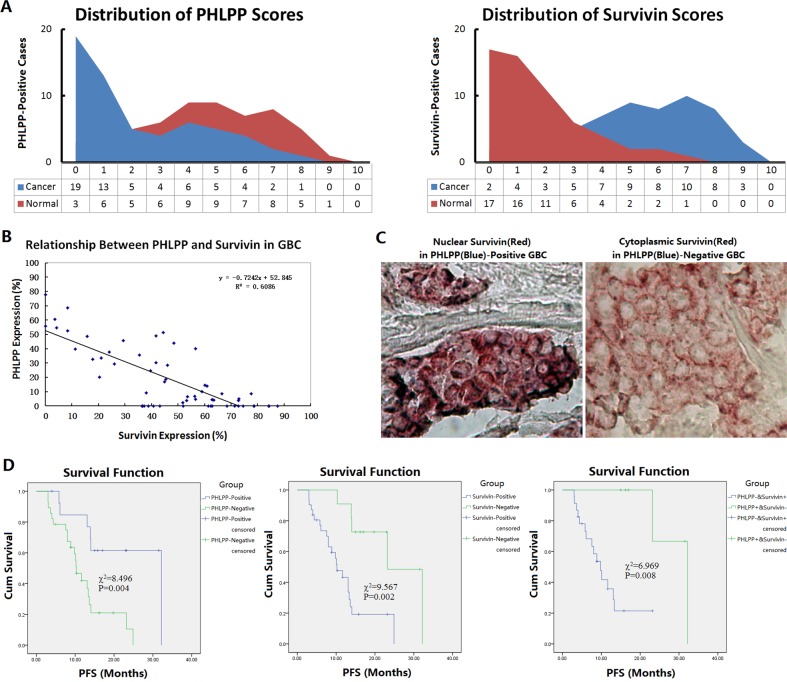
Expression of PHLPP and Survivin in GBC specimens and their relationships to prognosis in patients (**A**) Clinical specimens of GBC and gallbladder tissues were subjected to dual-staining immunohistochemistry to detect the localization and expression of PHLPP and Survivin. The percentages of positive cells were counted for all of the slices within five high-power fields under microscope, and the positive cell percentages from <10% to <100% were defined as score 1 to 10, all cells that were negative were scored as zero. The expression scores were showed in area diagram. (**B**) The expression levels of PHLPP and Survivin showed an inverse relationship. (**C**) Different intracellular localization of Survivin in PHLPP-positive and –negative GBC cells, which were showed blue and red staining visualized by 5-bromo-4-chloro-3-indolyl phosphate (BCIP)/tetranitroblue tetrazolium chloride (NBT) and 3-amino-9-ethylcarbazole (AEC), respectively, original magnification 400×. (**D**) The Kaplan-Meier analysis showed that both PHLPP and Survivin expression were closely associated with PFS in GBC patients, particularly the patients with PHLPP-negative and Survivin-positive had the worst prognosis in PFS.

The expression of PHLPP and Survivin in 59 cases of GBC specimens was detected by immunohistochemistry. The results showed that 30.51% (18/59) of GBC tissues were positive for PHLPP and 76.27% (45/59) positive for Survivin. In contrast, in the corresponding adjacent gallbladder mucosa tissues, the positive rates of PHLPP and Survivin were 66.10% (39/59) and 15.25% (9/59), respectively. Compared with the adjacent tissues, PHLPP expression was decreased (*P*=0.0001) and Survivin was increased (*P* = 0.0000) in cancer tissues (Fig. 1A). The PHLPP positive rates were 22.22% (10/45) among the Survivin-positive cases, and 57.14% (8/14) among the Survivin-negative cases. The expression levels of these two proteins showed an inverse relationship (R = −0.7242; Fig. 1B). PHLPP was mainly expressed in cytoplasm of cancer cells, and Survivin was mainly expressed in cytoplasm and nuclei of cancer cells. In the 45 Survivin-positive cases of GBC, 24 cases showed positive staining in both nuclei and cytoplasm (53.33%), 12 cases showed positive staining only in nuclei (26.67%) and 9 cases only in cytoplasm (20.00%). The 45 Survivin-positive GBC cases were divided into the PHLPP-positive (10 cases) and PHLPP-negative (35 cases) groups. Among the cases in the PHLPP-positive group, the Survivin-positive staining was observed in nuclei (5 cases), nucleocytoplasm (4 cases) and cytoplasm (1 case). The nuclear-positive cases were 90.00% (including nuclear-cytoplasmic positive and nuclear positive alone), more than the cytoplasmic-positive cases (50.00%, including nucleocytoplasmic positive and cytoplasmic positive alone). Among the cases in the PHLPP-negative group, the Survivin-positive staining was observed in nuclei (5 cases), nucleocytoplasm (9 cases) and cytoplasm (21 cases). The nuclear-positive cases were 40.00% (including nucleocytoplasmic positive and nuclear positive alone), fewer than the cytoplasmic-positive cases (85.71%, including nuclear-cytoplasmic positive and cytoplasmic positive alone) (Fig. 1C). The intracellular localizations of Survivin were significantly different between PHLPP-positive and PHLPP-negative groups. These results suggested that PHLPP may regulate Survivin intracellular localization.

The relationships between the clinicopathological features of GBC and the expression of Survivin and PHLPP were analyzed. The results showed that the decreased PHLPP expression was associated with local metastasis and late-stage of GBC, the increased Survivin expression was associated with local metastasis, late-stage and histological differentiation of GBC (Table 1). Among the 59 GBC patients, 42 patients had complete follow-up data. The follow-up times ranged from 3 months to 32 months, with a median follow-up time of 11 months. The median progression-free survival (PFS) was 6 months. Within the follow-up period, 15 patients had recurrence or progression, and 11 patients died. The Kaplan-Meier analysis showed that both PHLPP and Survivin expression were closely associated with PFS (PHLPP: 95% CI 10.73-16.01, *P* = 0.004; Survivin: 95% CI 10.72-16.02, *P* = 0.002). There was also a very significant difference in PFS between the PHLPP-negative & Survivin-positive patients and the PHLPP-positive & Survivin-negative patients (95% CI 8.29-17.85, *P* = 0.008; Fig. 1D).

**Table 1:** Relationship between the clinical parameters and the expressions of Survivin and PHLPP in GBC

Clinical parameters	n	PHLPP-Positive	*P* value	Survivin-Positive	*P* value
Age			0.6317		0.4935
≥ 60 year	30	10		24	
< 60 year	29	8		21	
Gender			0.7197		0.9665
Male	25	7		19	
Female	34	11		26	
Pathohistological Type			0.5555		0.3604
Infiltrating lesion	23	6		19	
Mass lesion	36	12		26	
Differentiation			0.3518		**0.0117**
Poor-medium	34	12		30	
Well	25	6		15	
Clinical Stage			**0.0343**		**0.0073**
I-II	24	11		14	
III-IV	35	7		31	
Metastasis			**0.0449**		**0.0255**
Positive	28	5		25	
Negative	31	13		20	
CA19-9			0.1722		0.1840
>37 U/ml	42	15		34	
≤37 U/ml	17	3		11	

### PHLPP induces GBC cell apoptosis and inhibits cell proliferation

**Figure 2:**
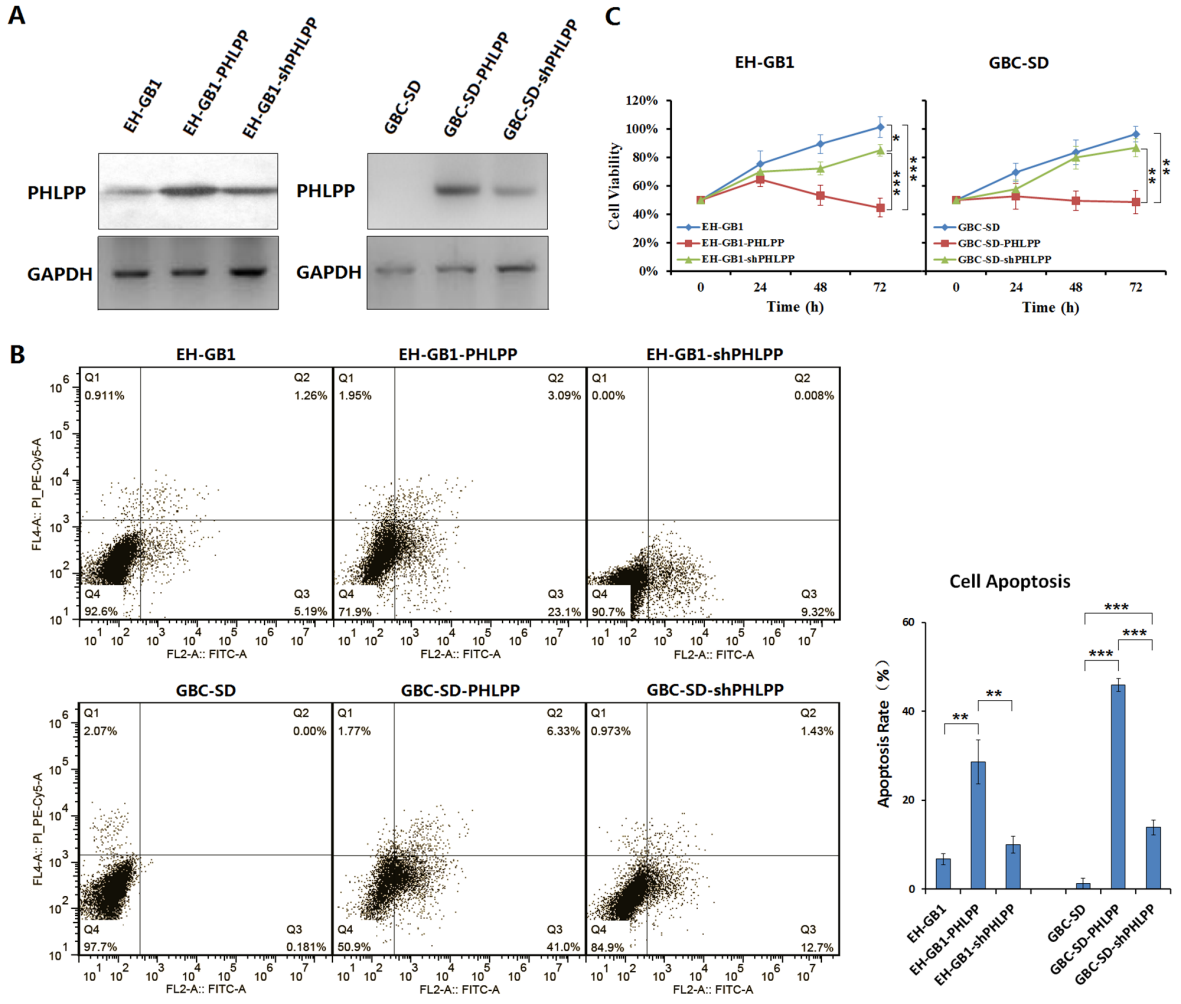
PHLPP induces apoptosis and inhibits proliferation in GBC cells (**A**) The EH-GB1 and GBC-SD cell lines were cultured, and 1 × 10^6^ cells at 24-well plates were infected with adenovirus Ad5-PHLPP (EH-GB1-PHLPP and GBC-SD-PHLPP), and then infected with adenovirus Ad5-shPHLPP (EH-GB1-shPHLPP and GBC-SD-shPHLPP), at a multiplicity of infection (MOI) of 100 pfu/cell. PHLPP expression was examined by Western blot. Glyceraldehyde-3-phosphate dehydrogenase (GAPDH) was used as an internal control. (**B**) The parental GBC cells and their sublines were stained with Annexin V / propidium iodide (PI), and cell apoptosis was measured with flow cytometer. The percentages of apoptotic cells, including the early and late apoptotic cells, were showed in histogram. **P < 0.01; ***P < 0.001. (**C**) The cell viability of GBC parental cells and cell sublines at 24 h, 48 h and 72 h was measured with the Cell Proliferation Kit in 96-well plates at 1 × 10^4^ cells/well. *P < 0.05; **P < 0.01; ***P < 0.001.

GBC cell lines EH-GB1 and GBC-SD were infected with adenovirus Ad5-PHLPP, and the PHLPP-overexpressing cell sublines were named EH-GB1-PHLPP and GBC-SD-PHLPP. The cell sublines were then transfected with Ad5-shPHLPP to knockdown PHLPP expression, and the PHLPP-silenced cell sublines were named EH-GB1-shPHLPP and GBC-SD-shPHLPP (Fig. 2A). The apoptosis was detected by flow cytometry in the EH-GB1 and GBC-SD parental cell lines and the cell sublines. It was found that overexpression of PHLPP significantly increased apoptosis in EH-GB1-PHLPP cells (*P* = 0.0017 versus EH-GB1) and GBC-SD-PHLPP cells (*P* = 0.0000 versus GBC-SD). Knockdown of PHLPP decreased the apoptosis rates in EH-GB1-shPHLPP cells (*P* = 0.0037 versus EH-GB1-PHLPP) and GBC-SD-shPHLPP cells (*P* = 0.0005 versus GBC-SD, *P*=0.0000 versus GBC-SD-PHLPP), suggesting that PHLPP has an activity of apoptosis induction in cancer cells (Fig. 2B). With the cell proliferation experiments, we found that the viabilities of EH-GB1-PHLPP and GBC-SD-PHLPP cells were significantly decreased and those of EH-GB1-shPHLPP and GBC-SD-shPHLPP cells were increased, suggesting that PHLPP has the function to inhibit cancer cell proliferation (Fig. 2C).

### PHLPP inhibits Survivin phosphorylation and promotes its nuclear translocation

**Figure 3:**
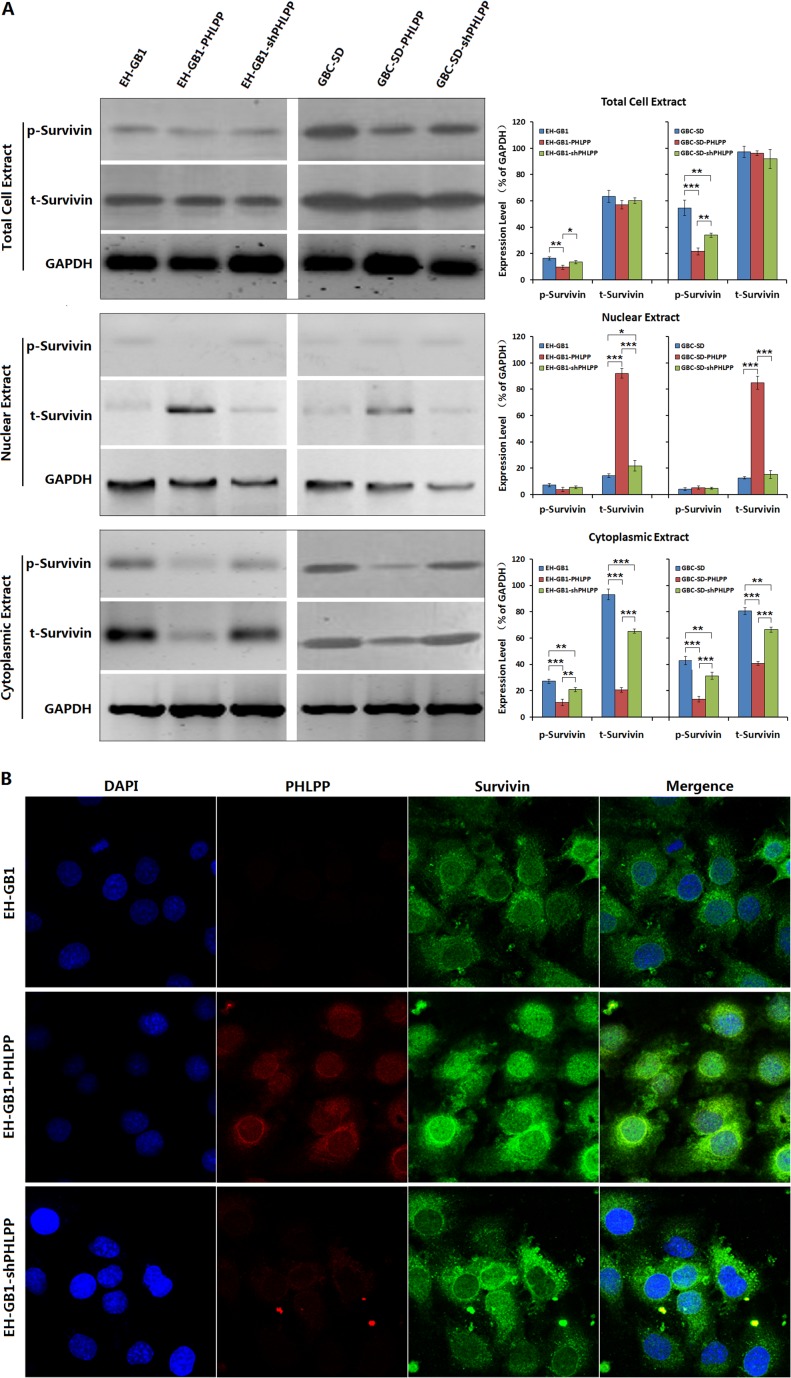
PHLPP inhibits Survivin phosphorylation and nuclear export (**A**) Total protein, cytoplasmic protein and nuclear protein were extracted from 1 × 10^6^ cells of the parental EH-GB1 and GBC-SD cell lines and their cell sublines, and the total Survivin (t-Survivin) and phosphorylated Survivin (p-Survivin) were examined by Western blot, with GAPDH as the loading control. The densitometry analysis was performed to show the relative expression levels normalized with GAPDH density. *P < 0.05; **P < 0.01; ***P < 0.001. (**B**) The EH-GB1 and its cell sublines were cultured into Lab-Tek chambers at 1 × 10^4^ cells/well, fixed in 4% formaldehyde for 30 min, and incubated with the primary antibodies at working concentration 1:200 at 4°C overnight, and the TRITC-conjugated or FITC-conjugated secondary antibodies at working concentration 1:500 at room temperature for 1 h. The cells were counterstained with dihydrochloride (DAPI) for nuclear background staining and observed under laser-scanning confocal microscope.

Survivin regulates apoptosis and cell cycle progression. These functions are linked to not only its intracellular localization but also its phosphorylation [20]. Therefore, we extracted cytoplasmic and nuclear protein from EH-GB1 and GBC-SD cells and the cell sublines to detect the phosphorylation levels and intracellular localization of Survivin by Western blotting and confocal imaging. The results showed that the phosphorylated Survivin (p-Survivin) in the EH-GB1-PHLPP and GBC-SD-PHLPP cells was decreased, particularly the cytoplasmic p-Survivin was decreased, and the nuclear p-Survivin maintained at a low level without change. The total amount of Survivin (t-Survivin) in the EH-GB1-PHLPP and GBC-SD-PHLPP cells did not change significantly compared with their parental cells, however, t-Survivin was increased in nuclei and decreased in cytoplasm. With PHLPP knockdown in the EH-GB1-shPHLPP and GBC-SD-shPHLPP cells, increases in p-Survivin levels mainly occurred in cytoplasm, while nuclear p-Survivin levels did not change. The nuclear t-Survivin was reduced and the cytoplasmic t-Survivin was significantly increased (Fig. 3A).

Confocal observations showed that the EH-GB1 cells were positive for Survivin expression, which mainly distributed in cytoplasm. With PHLPP overexpression, the cytoplasmic Survivin was decreased, while the nuclear Survivin was increased significantly. After re-knockdown of PHLPP expression, the nuclear Survivin was translocated again into cytoplasm (Fig. 3B). The results further demonstrated that PHLPP can regulate the intracellular localization of Survivin.

### The AKT signaling pathway is involved in PHLPP regulation of Survivin phosphorylation

**Figure 4:**
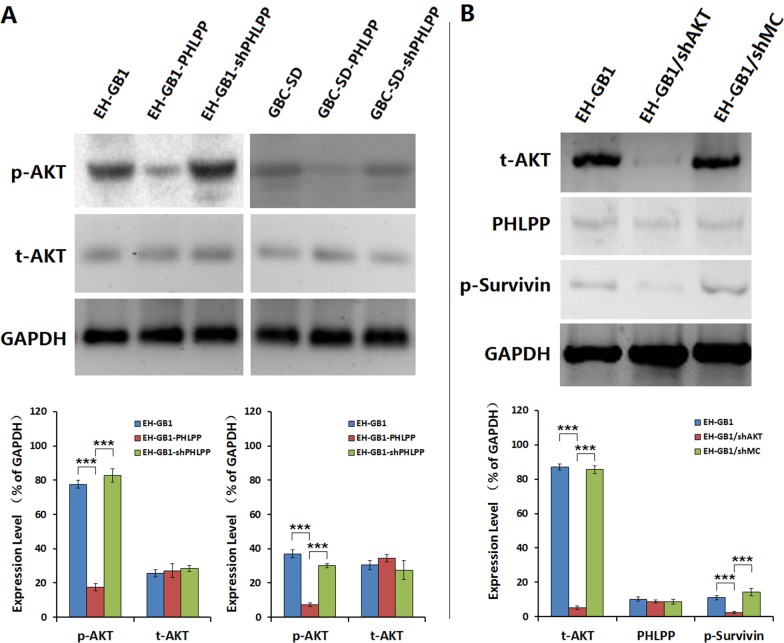
PHLPP inhibits Survivin phosphorylation through AKT signaling pathway (**A**) The expressions of total AKT (t-AKT) and phosphorylated AKT (p-AKT) were examined in 1 × 10^6^ cells of the parental EH-GB1 and GBC-SD cell lines and their cell sublines by Western blot, with GAPDH as the loading control. The densitometry analysis was performed to show the relative expression levels normalized with GAPDH density. ***P < 0.001. (**B**) The EH-GB1 cells were transfected with AKT shRNA vector (shAKT) and mock control shRNA vector (shMC), and the expressions of t-AKT, PHLPP and p-Survivin were examined by Western blot, with GAPDH as the loading control. The densitometry analysis was performed to show the relative expression levels normalized with GAPDH density. ***P < 0.001.

PHLPP can inhibit the activity of AKT signaling pathway [21]. Our previous study found that inhibiting the activity of AKT in cancer cells can reduce the phosphorylation of Survivin and downregulate Survivin expression, ultimately inducing cancer cells to undergo anoikis [22]. Therefore, we hypothesized that in GBC cells, the roles of PHLPP to inhibit Survivin phosphorylation and induce apoptosis may be related to the AKT signaling pathway. We determined the AKT phosphorylation levels in EH-GB1 and GBC-SD cells as well as in the cell sublines. We found that the phosphorylated AKT (p-AKT) levels were decreased in the EH-GB1-PHLPP and GBC-SD-PHLPP cells. Knocking down PHLPP again restored the p-AKT levels in the EH-GB1-shPHLPP and GBC-SD-shPHLPP cells (Fig. 4A). We knocked down AKT in EH-GB1 cells and found that downregulation of AKT did not affect the expression of PHLPP but decreased the levels of p-Survivin (Fig. 4B). The results suggested that PHLPP is an upstream regulatory factor of AKT and that AKT is involved in PHLPP-mediated regulation of Survivin phosphorylation in GBC cells.

### Decreased PHLPP levels was associated with high miR-495 expression in GBC cells

**Figure 5:**
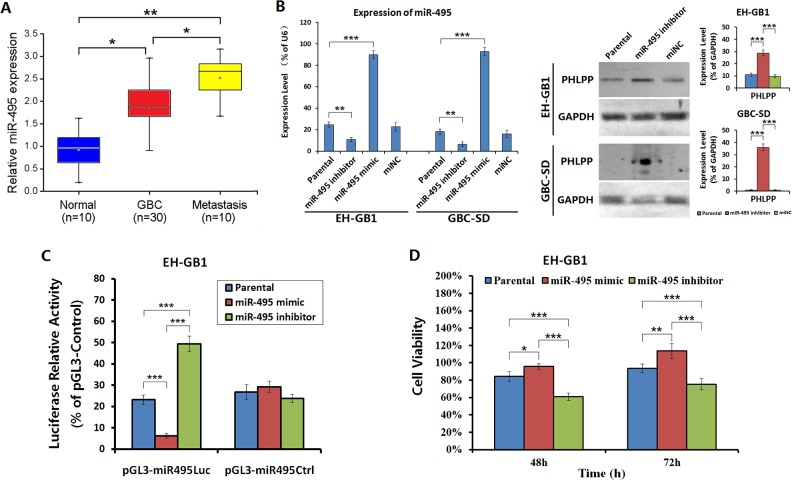
PHLPP was regulated by miR-495 in GBC cells (**A**) The expression of miR-495 in 30 cases of GBC primary tumor tissues, 10 cases of GBC liver metastatic tissues and 10 cases of normal gallbladder mucosa tissues was examined by qRT-PCR. *P < 0.05; **P < 0.01. (**B**) The EH-GB1 and GBC-SD cells were transfected with miR-495 mimic and miR-495 inhibitor, with miNC as the negative control. After confirming miR-495 expression levels, the PHLPP expression was detected by Western blot, with GAPDH as the loading control. The densitometry analysis was performed to show the relative expression levels normalized with GAPDH density. ***P < 0.001. (**C**) The regulation of miR-495 on PHLPP expression was measured by luciferase reporter assay. EH-GB1 and GBC-SD cells at 1 × 10^6^ cells/well in 24-well plates were transfected with miR-495 mimic or miR-495 inhibitor at 20 μg/well and cultured for 24 h. The wells were co-transfected with 20 ng pRL-TK together with 200 ng pGL3-miR495Luc, pGL3-miR495Ctrl, or pGL3-Control. The luciferase activity was measured 48 h later. The positive control vector pGL3-Control was used to normalize the relative activities of pGL3-miR495Luc and pGL3-miR495Ctrl. ***P < 0.001. (**D**) The aforementioned cells transfected with miR-495 mimic or miR-495 inhibitor were measured their viability at 48 h and 72 h after transfection. *P < 0.05; **P < 0.01; ***P < 0.001.

PHLPP can induce apoptosis in cancer cells and inhibit tumor growth, but PHLPP expression was decreased in GBC, and such a decrease is associated with poor prognosis of patients. Currently, the mechanism that results in the decrease of PHLPP in GBC is unknown. Using miRNA target gene prediction software (miRanda, TargetScan, picTar), we found that the 3′-UTR of PHLPP contains a seed sequence that combines miR-495 (GTTTGTT). We hypothesize that PHLPP may be a potential target of miR-495. Using qRT-PCR, we detected the miR-495 expression level in 30 cases of GBC primary tumor tissues, 10 cases of GBC liver metastatic tissues and 10 cases of normal gallbladder mucosa tissues. We found that miR-495 expression was significantly higher in GBC tissues than in normal tissues, and was higher in GBC metastatic tissues than in primary tumors (Fig. 5A). The miR-495 levels were high in both EH-GB1 and GBC-SD cells. Treatment of cells with miR-495 inhibitor resulted in increased PHLPP levels (Fig. 5B). The 3′-UTR of PHLPP was cloned and used to construct a luciferase reporter gene system. After transfected with miR-495 mimic in EH-GB1 and GBC-SD cells, the luciferase activity was decreased. In contrast, cells transfected with miR-495 inhibitor had an increased luciferase activity (Fig. 5C). Compared the cell proliferation activities of miR-495 mimic-transfected cells and miR-495 inhibitor-transfected cells, we found that the viability of miR-495 mimic-transfected cells was significantly higher than that of miR-495 inhibitor-transfected cells both at 48 h and 72 h after transfection (Fig. 5D). The results suggested that miR-495 is exactly an upstream regulator of PHLPP. High expression of miR-495 in GBC cells may be a major mechanism of PHLPP inactivation and increased proliferation of cancer cells.

### Anticancer activity of PHLPP gene therapy in GBC xenograft tumors in mice

**Figure 6:**
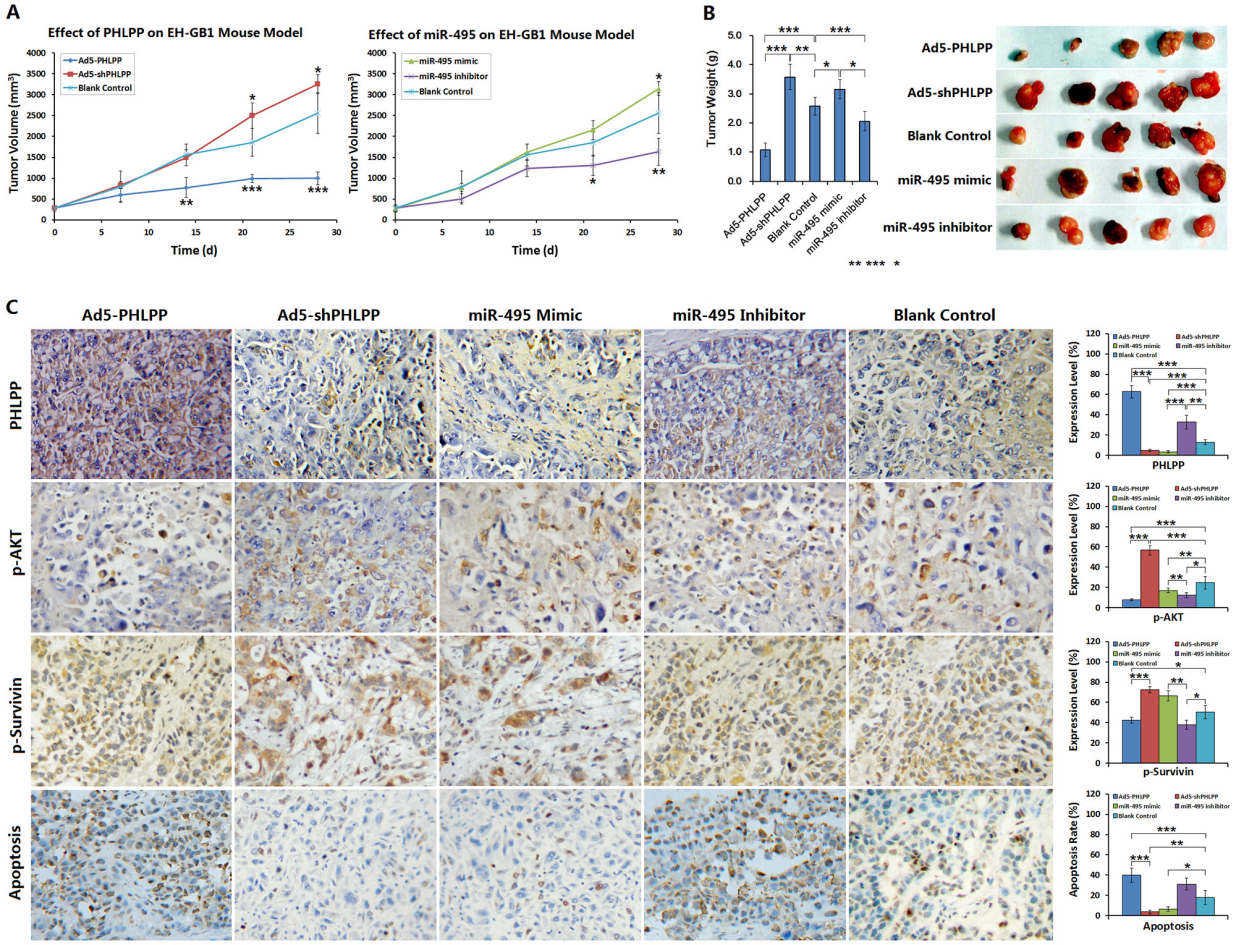
Effect of PHLPP or miR-495 on mouse GBC xenograft model (**A**) Nude mice were implanted with 5 × 10^6^ cells / mouse of EH-GB1 cells, and divided into five groups after forming xenograft tumors 12 days later. Each mouse received a total dose of 1 × 10^9^ pfu adenovirus, or 50 μg miR-495 mimic and miR-495 inhibitor. The tumor diameters were measured weekly and the tumor volumes were calculated. *P < 0.05; **P < 0.01; ***P<0.001 compared with the blank control group. (**B**) The xenograft tumors were collected and weighed. *P < 0.05; **P < 0.01; ***P < 0.001. (**C**) The xenograft tumors were fixed in 10% neutral buffered formalin for 6 h, paraffin-embedded, sectioned and prepared for detecting the expressions of PHLPP, p-AKT and p-Survivin by immunohistochemistry, and the cell apoptosis rates were detected by TUNEL assay. *P < 0.05; **P < 0.01; ***P < 0.001.

With EH-GB1 xenograft model in nude mice, we studied the effects of interfering with the miR-495/PPHLP1/AKT/Survivin pathway on GBC xenograft tumors. BALB/C mice were inoculated with EH-GB1 cells subcutaneously under the right arm. Once the tumors were formed, experiments interfering with PHLPP and miR-495 were performed. After the intervention therapy, within 14 days, tumors in the Ad5-PHLPP treatment group showed significant growth inhibition compared with the control group; within 21 days, tumors in the miR-495 inhibitor group showed growth inhibition, whereas tumors in the Ad5-shPHLPP group and the miR-495 mimic group had accelerated growth (Fig. 6A).

The xenografts were observed for 28 days after treatment. The observation was terminated when the tumor exceeded the limit defined by the Ethics Committee criteria (the maximal diameter was limited within 2 cm in any animal). The tumors were removed and weighed. Compare with the control group, the weights of the tumors in the Ad5-PHLPP group and in the miR-495 inhibitor group were significantly lower, and the weights of the tumors in the Ad5-shPHLPP group and in the miR-495 mimic group were significantly higher (Fig. 6B). Imunohistochemistry and TdT-mediated dUTP nick end labeling (TUNEL) were used to localize the expression of PHLPP, p-AKT, p-Survivin and to label apoptotic cells, respectively. Compared with those of the control group, the levels of p-AKT and p-Survivin were decreased in the Ad5-PHLPP group, and the apoptosis rate was increased. The p-AKT levels in tumors of the Ad5-shPHLPP group increasingly returned to that of the control group, the p-Survivin levels in Ad5-shPHLPP group were also increased but did not reach the levels of the control group, and the apoptosis rate was significantly decreased. In tumors of the miR-495 inhibitor group, the p-AKT and p-Survivin levels were decreased, and the apoptosis rate was increased. In tumors of the miR-495 mimic group, the p-AKT and p-Survivin levels were increased slightly but were not different compared with those of the control group, while the apoptotic rate was decreased (Fig. 6C).

## DISCUSSION

GBC is primarily originated in the gallbladder and cystic duct, and accounts for the most frequent malignancies in the biliary system with high degree of malignancy. Due to its high activity of proliferation and metastasis as well as lack of specific diagnostic markers, it is hard to detect the disease at early stage, therefore, the prognosis is often poor [1, 23]. In-depth study and screening of GBC molecular mechanisms and molecular targets, and establishment and optimization of comprehensive treatment strategies are some important aspects of improving the prognosis of patients with GBC. Recent studies have found that, similar to the majority of tumors, the tumorigenesis and progression of GBC involve a multi-factor, multi-step, multi-stage process, in which apoptosis negatively regulates the proliferation balance of cancer cells. The inhibition of apoptosis is one of the important causes of high proliferative activity in GBC [24, 25]. Apoptosis is closely correlated with the degree of histologic differentiation of GBC. With low levels of cancer cell differentiation and increased activity of proliferation and metastasis, the apoptosis index decreases, which confirms that apoptosis is an important mechanism involved in the regulation of biological behaviors of GBC. There are many factors involved in the regulation of apoptosis of GBC, among which Survivin and PHLPP are a pair of apoptotic regulators with opposite roles. Survivin has a strong inhibitory effect on apoptosis; in contrast, PHLPP can induce apoptosis. There are many questions that require further study regarding the roles of these two factors in the tumorigenesis and progression of GBC and molecular regulatory mechanisms of apoptosis.

High expression of Survivin in cancer patients is a valuable diagnostic and prognostic indicator. By inhibiting apoptosis and promoting cell cycle progression, high expression of Survivin enhances GBC invasion and metastasis [26]. The function of Survivin is relevant to its intracellular localization and phosphorylation status. Only when Survivin is exported to cytoplasm from nucleus, it can be phosphorylated and activated by protein kinases and exerts biological functions [27, 28]. A high level of cytoplasmic Survivin expression is considered a marker of poor prognosis, while the accumulation of nuclear Survivin is a marker of good prognosis [29]. In contrast to Survivin, PHLPP dephosphorylates and inactivates the protein kinases AKT and PKC and induces apoptosis in cancer cells. As such, the processes of phosphorylation and dephosphorylation of intracellular protein kinases are a key link to transformation of cell apoptosis and proliferation. Survivin and PHLPP possibly achieve mutual regulation through these processes. We studied PHLPP and Survivin expression in 59 GBC surgical specimens and found high expression levels of Survivin and low expression levels of PHLPP. These levels were inversely parallel and were closely related to the patients’ PFS. Both high Survivin expression and low PHLPP expression are important indicators of poor prognosis in GBC patients. Of note, we also found that in PHLPP-positive cancer tissues, Survivin-positive staining was mainly in nuclei, whereas in PHLPP-negative cancer tissues, Survivin-positive staining was mainly in cytoplasm. These results suggested that PHLPP may participate in the regulation of the intracellular localization of Survivin and may affect Survivin activity and function.

To clarify the common molecular mechanisms of PHLPP and Survivin in regulating apoptosis, we overexpressed PHLPP in the GBC cell lines EH-GB1 and GBC-SD and then knocked down its expression, followed by observing cell proliferation, apoptosis, Survivin phosphorylation levels and Survivin intracellular localization. Survivin phosphorylation can occur at threonine 34 (Thr34), threonine 53 (Thr53), threonine 117 (Thr117), serine 20 (Ser20) and many other sites. Survivin contains a Baculovirus IAP Repeat (BIR) domain specific to the inhibition of apoptosis proteins (IAP). Thr34 is exactly in this domain. Therefore, Survivin phosphorylation on Thr34 functions to protect cells by inhibiting apoptosis [20, 30]. The process of survivin phosphorylation by protein kinases occurs mainly in cytoplasm, so that the phosphorylated survivin concentrates in cytoplasm. After overexpressing PHLPP, the total Survivin transferred into and accumulated in nuclei and avoided to be phosphorylated, then both of the phosphorylated and total Survivin were decreased in cytoplasm of GBC cells. With the loss of Survivin function, cell apoptosis was enhanced while cell proliferation activity was reduced. The opposite effects were observed following knockdown of PHLPP expression. These results further demonstrated that PHLPP is an upstream regulatory factor of Survivin and induces apoptosis in GBC cells through inhibition of Survivin Thr34 phosphorylation and through promotion of nuclear translocation. Our previous study found that the AKT signal transduction pathway could be inhibited by p16 reactivation in cancer cells. This inhibition reduced the phosphorylation levels of Survivin and eventually induced cancer cell detachment-induced apoptosis, anoikis [22]. Because PHLPP can also dephosphorylate and inactivate AKT [21], we proposed that the functions of PHLPP, including inhibition of Survivin phosphorylation, promotion of Survivin nuclear accumulation and induction of apoptosis in GBC cells, may be associated with the AKT signal pathway. AKT participates in the signal transduction of a variety of growth factors. Increased AKT activity in malignant tumors is associated with highly malignant clinical biological behaviors of tumors. By examining AKT phosphorylation levels in GBC cell lines, we found that PHLPP can inhibit AKT phosphorylation. Inhibiting AKT expression directly impacts the phosphorylation of Survivin. These results confirmed that AKT is involved in PHLPP regulation of Survivin phosphorylation in GBC cells.

The expression of a tumor suppressor gene, PHLPP, is missing or decreased in a variety of tumors. However, there are many questions on the related causes and mechanisms [19, 31]. The PHLPP 3′-UTR contains a seed sequence that combines miR-495. Many tumor tissues, including GBC, show high expression of miR-495 and low expression of PHLPP. Therefore, we hypothesized that PHLPP is a potential target of miR-495. Inhibiting the expression of miR-495 in GBC cells led to an increase of PHLPP expression. Luciferase reporter gene assay confirmed that miR-495 plays a regulatory role by binding to the PHLPP 3′-UTR. Increasing the expression of miR-495 in GBC cells enhanced cell proliferation, and inhibition of miR-495 expression in GBC cells decreased cell viability. These results suggested that PHLPP is a target gene of miR-495. By interfering with miR-495 and its target gene PHLPP in xenograft tumors in nude mice, we found that upregulating miR-495 expression or inhibiting PHLPP expression could increase phosphorylated AKT and phosphorylated Survivin levels, leading to inhibited apoptosis and enhanced growth of GBC xenograft tumors. Further, downregulation of miR-495 expression or increased PHLPP expression was able to reduce the level of phosphorylated AKT and phosphorylated Survivin, leading to the induction of apoptosis and inhibited tumor growth.

**Figure 7:**
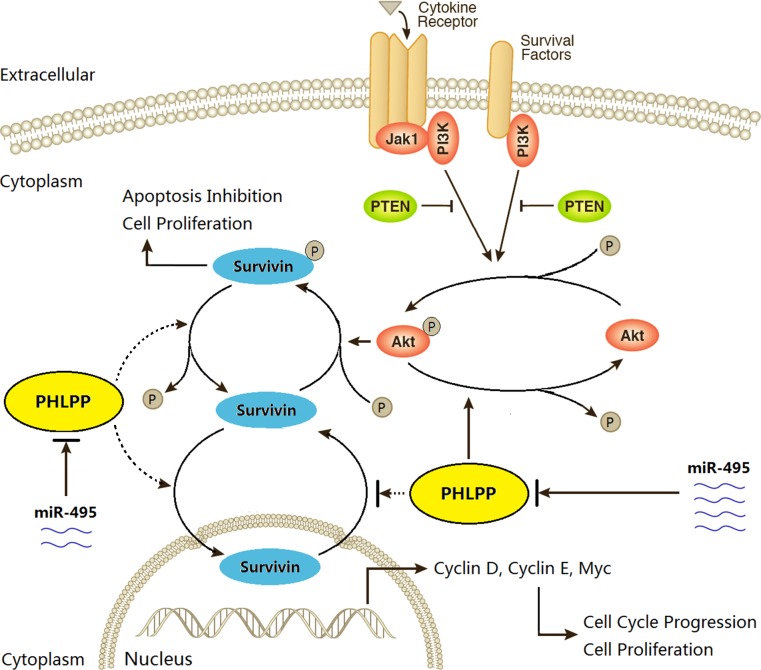
The schematic diagram of miR-495/PHLPP/AKT/Survivin regulatory pathway potentially existed in GBC The phosphorylation of AKT is a key step for regulating cell proliferation and apoptosis. The extracellular cytokines and survival factors activate AKT signaling, which promotes the phosphorylation of Survivin and finally results in apoptosis inhibition and cell proliferation. However, PHLPP, that is under the regulation of miR-495, may dephosphorylate AKT and inactivate AKT signaling pathway, and also possibly dephosphorylate Survivin and promotes Survivin transportation into nuclei, then inactivates the function of Survivin, finally results in an increase of cell apoptosis and decrease of cell proliferation.

In summary, our experiments showed that a miR-495 / PHLPP / AKT / Survivin pathway regulates the conversion between apoptosis and proliferation of GBC cells (Fig. 7). High expression of miR-495 in GBC tumor cells may be a major mechanism of PHLPP inactivation, cell resistance to apoptosis and enhanced proliferation. Gene therapy intervention specific for miR-495 and its target gene PHLPP may provide an effective alternative strategy to develop target therapies for GBC.

## MATERIALS AND METHODS

### GBC clinical specimens and follow-up of the patients

GBC tissues from 59 cases and the corresponding adjacent mucosa tissues were obtained from patients who received surgery between 2010 and 2012. The patients did not receive preoperative chemotherapy, radiotherapy or other methods of treatments. The subjects included 25 males and 34 females. The male to female ratio was 1:1.36. The patient ages were between 29 and 77, with a median age of 56 years. According to the pathological diagnosis, there were 34 cases of poorly differentiated adenocarcinoma, 25 cases of well-differentiated adenocarcinoma, and 19 cases of tumors with local (liver and hepatic hilar lymph node) metastasis, 23 cases showed infiltrating lesions and 36 cases showed mass lesions. According to the Union for International Cancer Control (UICC) standard clinical stage, there were 24 cases at stages I-II and 35 cases at stages III-IV, 42 cases with high levels of serum CA19-9 before operation. The specimens were fixed in 10% neutral buffered formalin for 6 h, paraffin-embedded, sectioned and prepared for immunohistochemical experiments for the detection of protein expression. Fresh tissues from 30 cases of primary tumor tissue GBC, 10 cases of liver metastasis GBC tissues and 10 cases of normal gallbladder mucosa tissues were preserved for detecting miR-495 expression levels by qRT-PCR. There were 42 cases of patients with complete follow-up data. The follow-up periods were from the time of surgery to June 30, 2014. The PFS time was calculated from the beginning of the follow-up period to disease recurrence or death.

### Construction of vectors and transfection of GBC cells

Using the human PHLPP sequence (GenBank: BC126277.1) as a reference, we constructed PHLPP expression adenovirus vector Ad5-PHLPP and PHLPP shRNA adenovirus vector Ad5-shPHLPP, according to the method described in the literature [32]. The shRNA vector of PHLPP contained 19 nucleotides targeting 181-199 bp of the PHLPP gene (5′-CTG AAC GGA TTC AGC TCT C-3′) and the shRNA vector of AKT containing 19 nucleotides targeting 1338-1356 bp of the Akt1 gene (NM_005163; 5′-GAC TAC CTG CAC TCG GAG A-3′) were constructed. A mock control shRNA (shMC; 5′-GAC TTC ATA AGG CGC ATG C-3′) and a control adenovirus Ad5-EGFP (containing the EGFP reporter gene) were synchronously constructed.

The EH-GB1 gallbladder cancer cell line was established by the Shanghai Eastern Hepatobiliary Surgery Hospital [33]. The GBC-SD cell line was received from the Shanghai Institute of Cell Biology, Chinese Academy of Sciences. Cells were cultured under the conditions suggested by the vendors and were infected with adenovirus at a multiplicity of infection (MOI) of 100 pfu/cell. Cells infected with Ad5-PHLPP were named the EH-GB1-PHLPP and GBC-SD-PHLPP cell sublines. Cells infected with Ad5-shPHLPP were named the EH-GB1-shPHLPP and GBC-SD-shPHLPP cell sublines.

### Animal experiments

Thirty 4-week-old healthy male purebred BALB/C mice were provided by Shanghai SLAC Experimental Animal Center of Chinese Academy of Sciences. EH-GB1 cells at the logarithmic growth phase were suspended and injected subcutaneously under the right arms of nude mice, at 5 × 10^6^ cells / 100 μl / animal. At 10 days after inoculation, the tumor formation rate was 100%. The diameters of the transplanted tumors were approximately 0.7-0.9 cm. Five animals whose tumors were too large or too small were excluded. The remaining 25 animals were randomly divided into five groups (Ad5-PHLPP, Ad5-shPHLPP, miR-495 mimic, miR-495 inhibitor, and blank control groups). Mice in the Ad5-PHLPP and Ad5-shPHLPP groups were given multi-point injections of corresponding adenoviruses into the tumors. Each mouse received a viral dose of 2 × 10^8^ pfu / 100 μl, once every other day, for a total of five times. Mice in the miR-495 mimic and miR-495 inhibitor groups received injections of miR-495 mimic and miR-495 inhibitor (Guangzhou RiboBio Co., Ltd., Guangzhou, China) of 10 μg / 100 μl / mouse, respectively, once every other day, for a total of five times. Mice in the control group received synchronously saline injections of 100 μl / mouse every time. After treatments, the tumor sizes were measured weekly, and the tumor volumes were determined as “minimum diameter × maximum diameter^2^ × 0.5”. At the end of observation, the mice were sacrificed under anesthesia. Tumor specimens were collected and weighed. The fresh tissues were fixed in 10% neutral buffered formalin, paraffin-embedded, sectioned and prepared for gene expression and apoptosis detection.

### Gene expression levels in GBC tissues and cell lines

The parental EH-GB1 and GBC-SD cell lines and their cell sublines were cultured for 48 h. Three samples of 1 × 10^6^ cells were collected and used for extracting total protein, cytoplasmic protein and nuclear protein by the Nuclear and Cytoplasmic Extraction Reagents (Pierce Biotechnology, Inc., Rockford, IL, USA) according to the manufacturer's instructions. The antibodies used in detection of gene expression by Western blotting included the rabbit anti-PHLPP (Abcam Company Ltd., Shanghai, China), rabbit anti-phosphorylated Survivin^Thr34^, mouse anti-AKT, rabbit anti-phosphorylated AKT^Thr308^ (Santa Cruz Biotechnology, Inc., Santa Cruz, CA, USA), mouse anti-Survivin (Invitrogen, Carlsbad, CA, USA).

The EH-GB1 and its cell sublines were seeded at 1 × 10^4^ cells/well into Lab-Tek chambers (Electron Microscopy Sciences, Hatfield, PA, USA). After 48 h, cells were fixed in 4% formaldehyde for 30 min, incubated with the primary antibodies (rabbit anti-PHLPP and mouse anti-Survivin; working concentration 1:200) at 4 °C overnight, then cultured with the secondary antibodies (TRITC-conjugated anti-rabbit IgG and FITC-conjugated anti-mouse IgG; working concentration 1:500; Santa Cruz Biotechnology, Inc., Santa Cruz, CA, USA) at room temperature for 1 h. The cells were counterstained with dihydrochloride (DAPI) for nuclear background staining. The staining was observed with laser-scanning confocal microscopy (Zeiss LSM510, Carl Zeiss Inc., Germany).

GBC clinical specimens and xenograft tumor tissues were paraffin-embedded, sectioned and subjected to streptavidin-peroxidase (SP) immunohistochemistry to detect the localization and expression of PHLPP, Survivin, p-AKT and p-Survivin. The percentages of positive cells were counted for all of the slices within five high-power fields under microscope, and the positive cell percentages from <10% to <100% were defined as score 1 to 10, all cells that were negative were scored as zero. Patient with 4 or more than score 4 was qualified as positive case.

### Apoptosis of GBC tissue and cell lines

GBC cells were cultured, and 1 × 10^6^ cells were collected. Annexin V / propidium iodide (PI) staining procedures were performed according to the following reference [34]. Apoptosis was detected with flow cytometer (FACS420, BD Biosciences, San Jose, CA, USA). Xenografted tumors in BALB/C nude mice were paraffin-embedded, sectioned and subjected to TUNEL assay (Maxim Biotech Inc., Fuzhou, China) to detect cell apoptosis [33]. The proportions of positive cells in each slice were counted within five high-power fields under microscope.

### Cell proliferation experiment

The Cell Proliferation Kit I (Roche Diagnostics GmbH, Germany) was used to detect the cell viability of the parental and cell sublines of EH-GB1 and GBC-SD at 24 h, 48 h and 72 h according to the methods in the literature [32, 35].

### miR-495 expression in GBC tissues and cell lines

miRNAs from 1 × 10^6^ GBC cells or 200 μg of GBC clinical specimens or xenograft tumor tissues were extracted using the mirVana miRNA Isolation Kit (Applied Biosystems, Foster City, CA, USA). The miR-495 expression was determined using the MiniOpticonTM Two-Color Real-Time PCR Detection System (Bio-Rad, Hercules, CA, USA), with U6 as an internal reference.

### Analysis of luciferase activity

Using the PHLPP downstream 3′-UTR sequence as a reference, we designed and constructed a pGL3-miR495Luc luciferase reporter gene plasmid. By mutating the complement sequences of the miR495 seed sequence in PHLPP 3′-UTR, we constructed a negative control vector pGL3-miR495Ctrl in which the miR-495 binding sites were mutated. The pGL3-Control vector (Promega Trading Co., Ltd., Shanghai, China) was used for a positive control vector. Next, 1 × 10^6^ EH-GB1 and GBC-SD cells were seeded in 24-well plates and transfected with miR-495 mimic or miR-495 inhibitor at 20 μg / well using Lipofectamine^TM^ 2000 (Invitrogen, Carlsbad, CA). Twenty-four hours after transfection, the wells were co-transfected with 20 ng of pRL-TK (Promega Trading Co., Ltd.) together with 200 ng of pGL3-miR495Luc, pGL3-miR495Ctrl, or pGL3-Control. Cells were collected at 48 h after transfection for extraction of total protein. The luciferase activity was measured according to the instructions of the Dual-Luciferase® Reporter Assay System Kit (Promega Trading Co., Ltd.). Using the control pGL3-Control vector as a standard, the relative luciferase activities of the pGL3-miR495Luc and pGL3-miR495Ctrl expression vectors were calculated.

### Statistical analyses

The experimental data from 3 times of independent *in vitro* experiments, as well as the in vivo experimental data from 5 mice per group, were presented as ‘mean ± standard deviation’, and analyzed by one-way analysis of variance (ANOVA). When the data were statistically different among the multiple groups, the SNK-q test was used to conduct the multiple comparisons. The clinicopathological parameters were evaluated by chi-square test, and the patients’ PFS was calculated by Kaplan-Meier method and compared through the log-rank test. The statistical analysis software was PASW Statistics 18. The *P* values less than 0.05 were considered statistically significant.

## References

[1] Nigam J, Chandra A, Kazmi HR, Singh A, Gupta V, Parmar D, Srivastava MK (2014). Expression of serum survivin protein in diagnosis and prognosis of gallbladder cancer: a comparative study. Med Oncol.

[2] Haydn JM, Hufnagel A, Grimm J, Maurus K, Schartl M, Meierjohann S (2014). The MAPK pathway as an apoptosis enhancer in melanoma. Oncotarget.

[3] Will M, Qin AC, Toy W, Yao Z, Rodrik-Outmezguine V, Schneider C, Huang X, Monian P, Jiang X, de Stanchina E, Baselga J, Liu N, Chandarlapaty S, Rosen N (2014). Rapid induction of apoptosis by PI3K inhibitors is dependent upon their transient inhibition of RAS-ERK signaling. Cancer Discov.

[4] Qin B, Ariyama H, Baba E, Tanaka R, Kusaba H, Harada M, Nakano S (2006). Activated Src and Ras induce gefitinib resistance by activation of signaling pathways downstream of epidermal growth factor receptor in human gallbladder adenocarcinoma cells. Cancer Chemother Pharmacol.

[5] Siveen KS, Nguyen AH, Lee JH, Li F, Singh SS, Kumar AP, Low G, Jha S, Tergaonkar V, Ahn KS, Sethi G (2014). Negative regulation of signal transducer and activator of transcription-3 signalling cascade by lupeol inhibits growth and induces apoptosis in hepatocellular carcinoma cells. Br J Cancer.

[6] Xiang SS, Wang XA, Li HF, Shu YJ, Bao RF, Zhang F, Cao Y, Ye YY, Weng H, Wu WG, Mu JS, Wu XS, Li ML, Hu YP, Jiang L, Tan ZJ, Lu W, Liu F, Liu YB (2014). Schisandrin B induces apoptosis and cell cycle arrest of gallbladder cancer cells. Molecules.

[7] Athanasoula KCh, Gogas H, Polonifi K, Vaiopoulos AG, Polyzos A, Mantzourani M (2014). Survivin beyond physiology: orchestration of multistep carcinogenesis and therapeutic potentials. Cancer Lett.

[8] Salzano G, Riehle R, Navarro G, Perche F, De Rosa G, Torchilin VP (2014). Polymeric micelles containing reversibly phospholipid-modified anti-survivin siRNA: a promising strategy to overcome drug resistance in cancer. Cancer Lett.

[9] Hartman ML, Czyz M (2013). Anti-apoptotic proteins on guard of melanoma cell survival. Cancer Lett.

[10] Altieri DC (2013). Targeting survivin in cancer. Cancer Lett.

[11] Santa Cruz Guindalini R, Mathias Machado MC, Garicochea B (2013). Monitoring survivin expression in cancer: implications for prognosis and therapy. Mol Diagn Ther.

[12] Trotman LC, Pandolfi PP (2003). PTEN and p53: who will get the upper hand?. Cancer Cell.

[13] Freeman DJ, Li AG, Wei G, Li HH, Kertesz N, Lesche R, Whale AD, Martinez-Diaz H, Rozengurt N, Cardiff RD, Liu X, Wu H (2003). PTEN tumor suppressor regulates p53 protein levels and activity through phosphatase-dependent and -independent mechanisms. Cancer Cell.

[14] Jia J, Li S, Gong W, Ding J, Fang C, Quan Z (2011). mda-7/IL-24 induces apoptosis in human GBC-SD gallbladder carcinoma cells via mitochondrial apoptotic pathway. Oncol Rep.

[15] Gao T, Furnari F, Newton AC (2005). PHLPP: a phosphatase that directly dephosphorylates Akt, promotes apoptosis, and suppresses tumor growth. Mol Cell.

[16] O'Neill AK, Niederst MJ, Newton AC (2013). Suppression of survival signalling pathways by the phosphatase PHLPP. FEBS J.

[17] Qiao M, Wang Y, Xu X, Lu J, Dong Y, Tao W, Stein J, Stein GS, Iglehart JD, Shi Q, Pardee AB (2010). Mst1 is an interacting protein that mediates PHLPPs’ induced apoptosis. Mol Cell.

[18] Nitsche C, Edderkaoui M, Moore RM, Eibl G, Kasahara N, Treger J, Grippo PJ, Mayerle J, Lerch MM, Gukovskaya AS (2012). The phosphatase PHLPP1 regulates Akt2, promotes pancreatic cancer cell death, and inhibits tumor formation. Gastroenterology.

[19] Liu J, Weiss HL, Rychahou P, Jackson LN, Evers BM, Gao T (2009). Loss of PHLPP expression in colon cancer: role in proliferation and tumorigenesis. Oncogene.

[20] Cheung CH, Huang CC, Tsai FY, Lee JY, Cheng SM, Chang YC, Huang YC, Chen SH, Chang JY (2013). Survivin - biology and potential as a therapeutic target in oncology. Onco Targets Ther.

[21] Newton AC, Trotman LC (2014). Turning off AKT: PHLPP as a drug target. Annu Rev Pharmacol Toxicol.

[22] Hu H, Li Z, Chen J, Wang D, Ma J, Wang W, Li J, Wu H, Li L, Wu M, Qian Q, Chen J, Su C (2011). P16 reactivation induces anoikis and exhibits antitumour potency by downregulating Akt/survivin signalling in hepatocellular carcinoma cells. Gut.

[23] Kazmi HR, Chandra A, Nigam J, Noushif M, Parmar D, Gupta V (2013). Prognostic significance of K-ras codon 12 mutation in patients with resected gallbladder cancer. Dig Surg.

[24] Ono M1, Higuchi T, Takeshima M, Chen C, Nakano S (2013). Antiproliferative and apoptosis-inducing activity of curcumin against human gallbladder adenocarcinoma cells. Anticancer Res.

[25] Rai R, Tewari M, Kumar M, Singh AK, Shukla HS (2011). p53: its alteration and gallbladder cancer. Eur J Cancer Prev.

[26] Nigam J, Chandra A, Kazmi HR, Parmar D, Singh D, Gupta V MN (2014). Expression of survivin mRNA in gallbladder cancer: a diagnostic and prognostic marker?. Tumour Biol.

[27] Knauer SK, Krämer OH, Knösel T, Engels K, Rödel F, Kovács AF, Dietmaier W, Klein-Hitpass L, Habtemichael N, Schweitzer A, Brieger J, Rödel C, Mann W, Petersen I, Heinzel T, Stauber RH (2007). Nuclear export is essential for the tumor-promoting activity of survivin. FASEB J.

[28] Stauber RH, Mann W, Knauer SK (2007). Nuclear and cytoplasmic survivin: molecular mechanism, prognostic, and therapeutic potential. Cancer Res.

[29] Qi G, Tuncel H, Aoki E, Tanaka S, Oka S, Kaneko I, Okamoto M, Tatsuka M, Nakai S, Shimamoto F (2009). Intracellular localization of survivin determines biological behavior in colorectal cancer. Oncol Rep.

[30] Barrett RM, Osborne TP, Wheatley SP (2009). Phosphorylation of survivin at threonine 34 inhibits its mitotic function and enhances its cytoprotective activity. Cell Cycle.

[31] Chen M, Pratt CP, Zeeman ME, Schultz N, Taylor BS, O'Neill A, Castillo-Martin M, Nowak DG, Naguib A, Grace DM, Murn J, Navin N, Atwal GS, Sander C, Gerald WL, Cordon-Cardo C, Newton AC, Carver BS, Trotman LC (2011). Identification of PHLPP1 as a tumor suppressor reveals the role of feedback activation in PTEN-mutant prostate cancer progression. Cancer Cell.

[32] Li Z, Li X, Li C, Su Y, Fang W, Zhong C, Ji W, Zhang Q, Su C (2014). Transcription factor OCT4 promotes cell cycle progression by regulating CCND1 expression in esophageal carcinoma. Cancer Lett.

[33] Liu C, Sun B, An N, Tan W, Cao L, Luo X, Yu Y, Feng F, Li B, Wu M, Su C, Jiang X (2011). Inhibitory effect of Survivin promoter-regulated oncolytic adenovirus carrying P53 gene against gallbladder cancer. Mol Oncol.

[34] Li C, Yan Y, Ji W, Bao L, Qian H, Chen L, Wu M, Chen H, Li Z, Su C (2012). OCT4 positively regulates Survivin expression to promote cancer cell proliferation and leads to poor prognosis in esophageal squamous cell carcinoma. PLoS One.

[35] Zhang Y, Fang L, Zhang Q, Zheng Q, Tong J, Fu X, Jiang X, Su C, Zheng J (2013). An oncolytic adenovirus regulated by a radiation-inducible promoter selectively mediates hSulf-1 gene expression and mutually reinforces antitumor activity of I131-metuximab in hepatocellular carcinoma. Mol Oncol.

